# Supplementary figures and images for: RNA N6-methyladenosine reader IGF2BP3 promotes acute myeloid leukemia progression by controlling stabilization of EPOR mRNA
Source: PeerJ. 2023 Aug 30;11:e15706. doi: 10.7717/peerj.15706 (PMC10474828; doi:10.7717/peerj.15706)

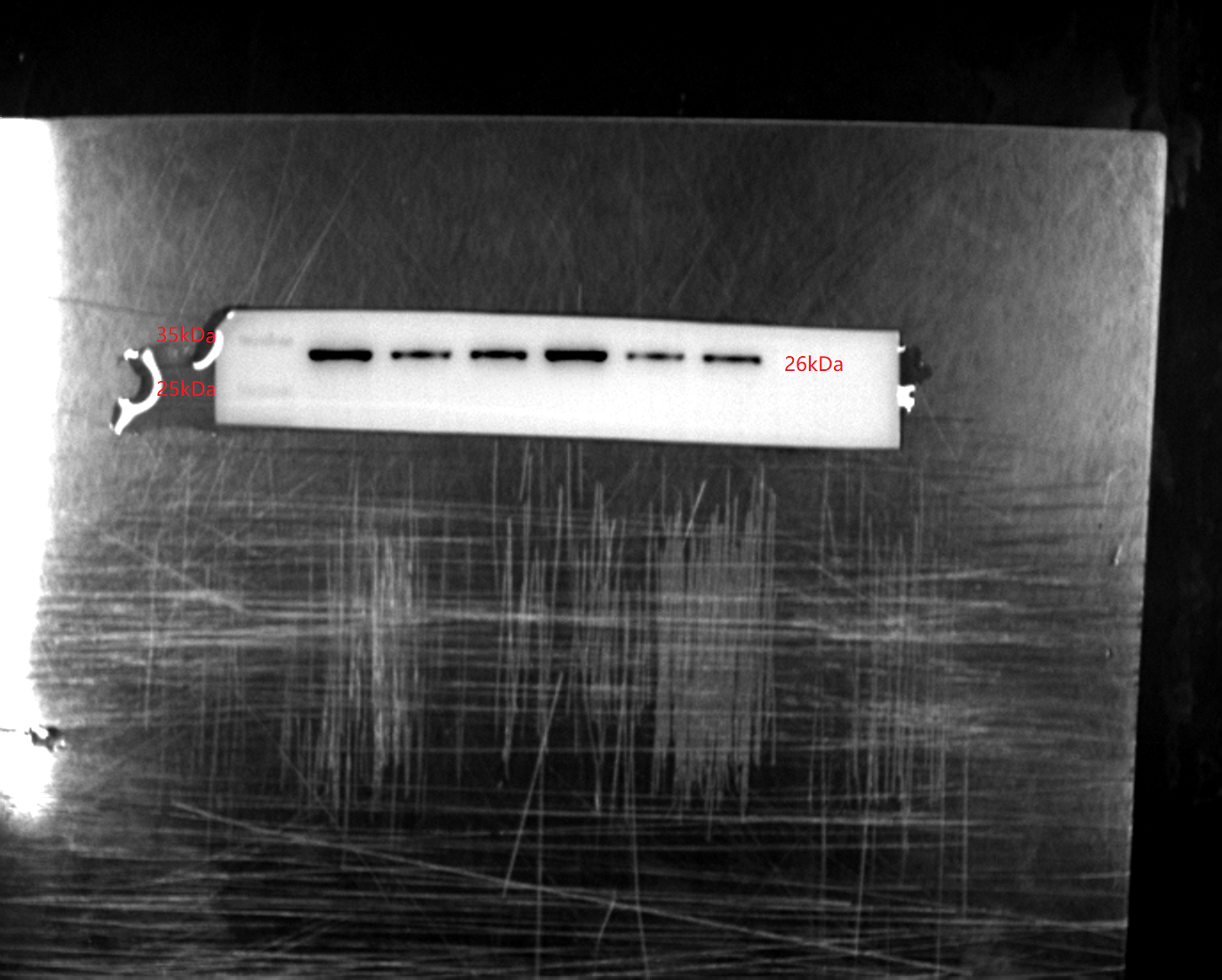

Supplement: Supplemental Information 1 [file peerj-11-15706-s001.zip › Full-length uncropped gelsblots/BCL-2.Tif]

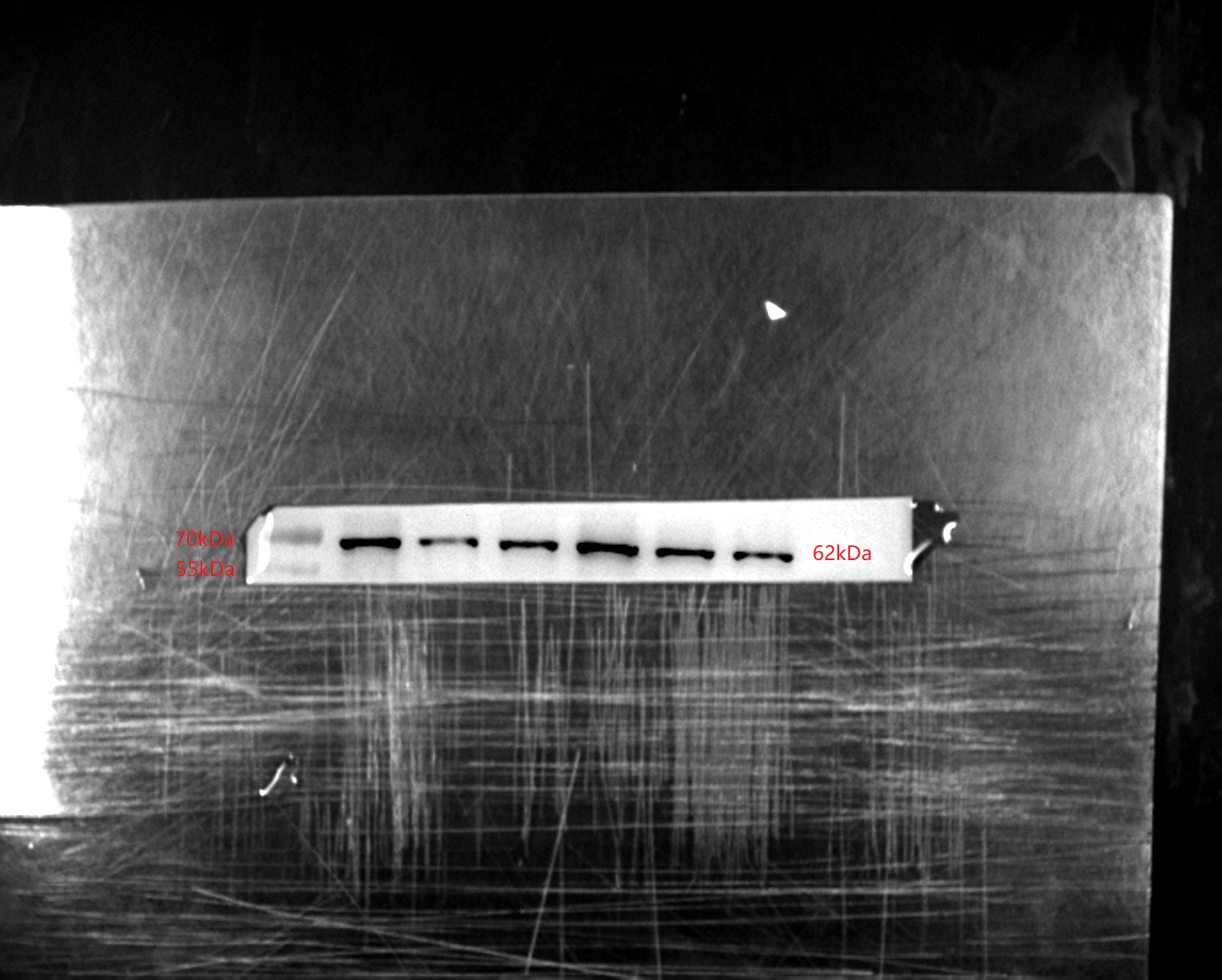

Supplement: Supplemental Information 1 [file peerj-11-15706-s001.zip › Full-length uncropped gelsblots/c-myc.Tif]

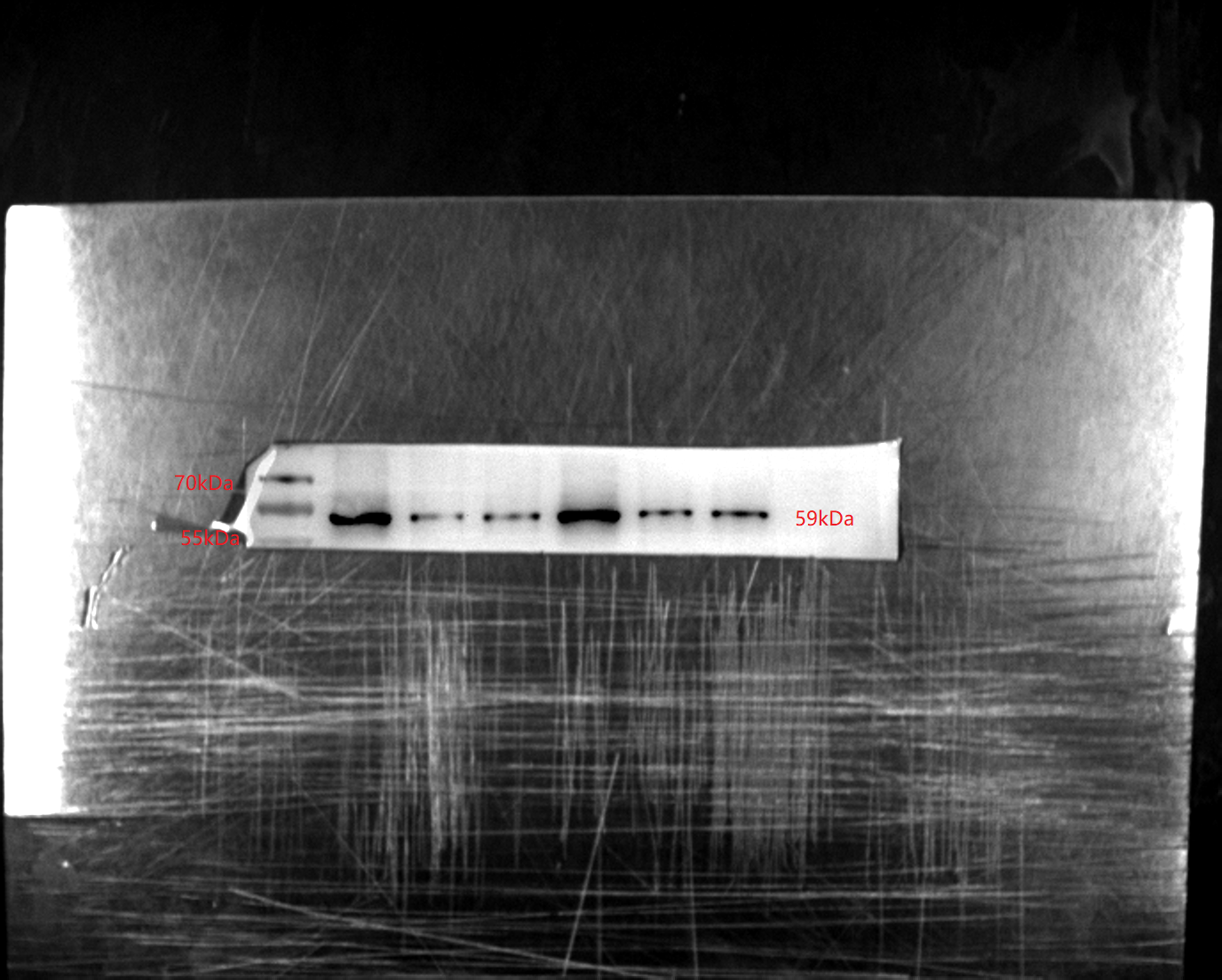

Supplement: Supplemental Information 1 [file peerj-11-15706-s001.zip › Full-length uncropped gelsblots/EPOR.Tif]

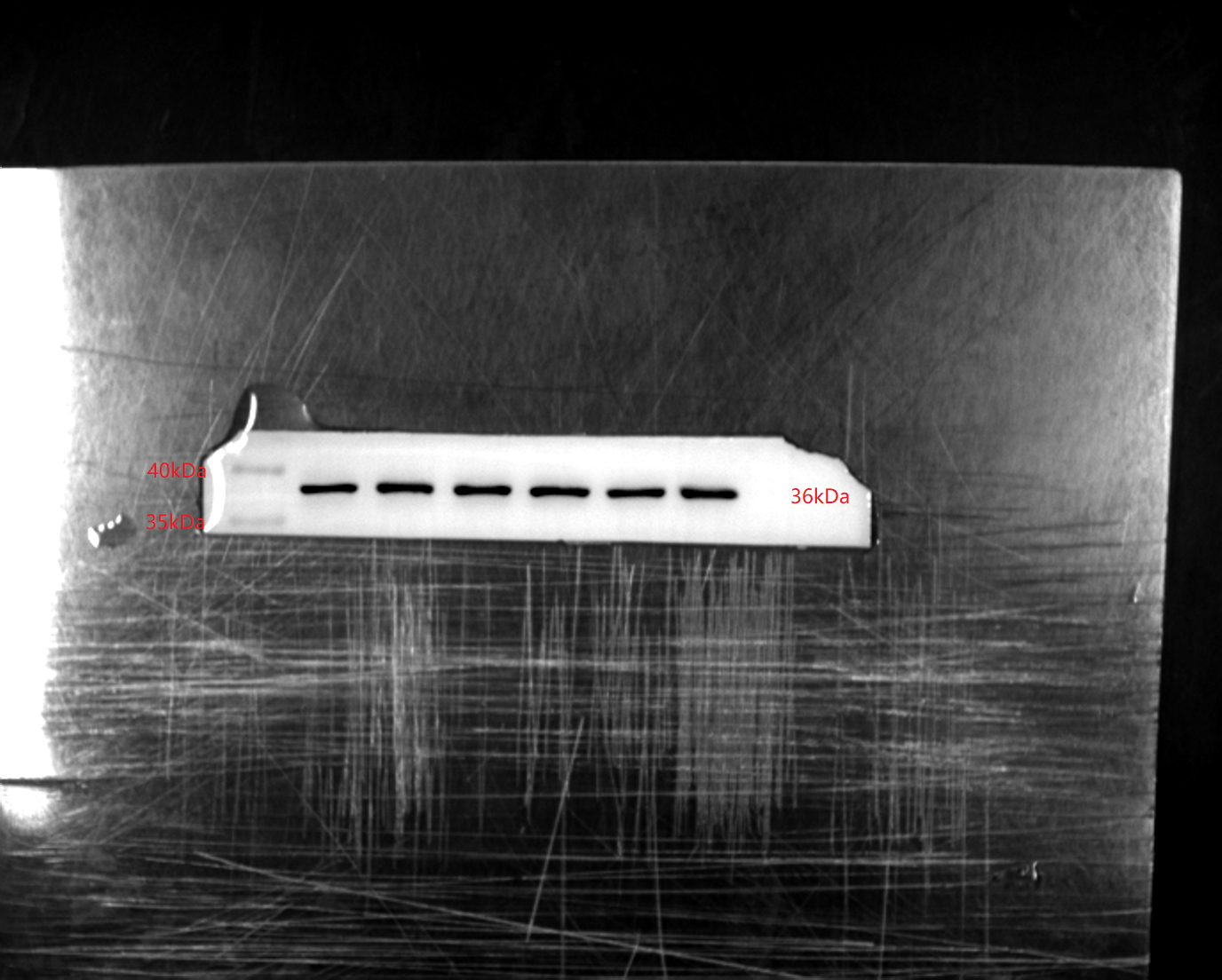

Supplement: Supplemental Information 1 [file peerj-11-15706-s001.zip › Full-length uncropped gelsblots/GAPDH.Tif]

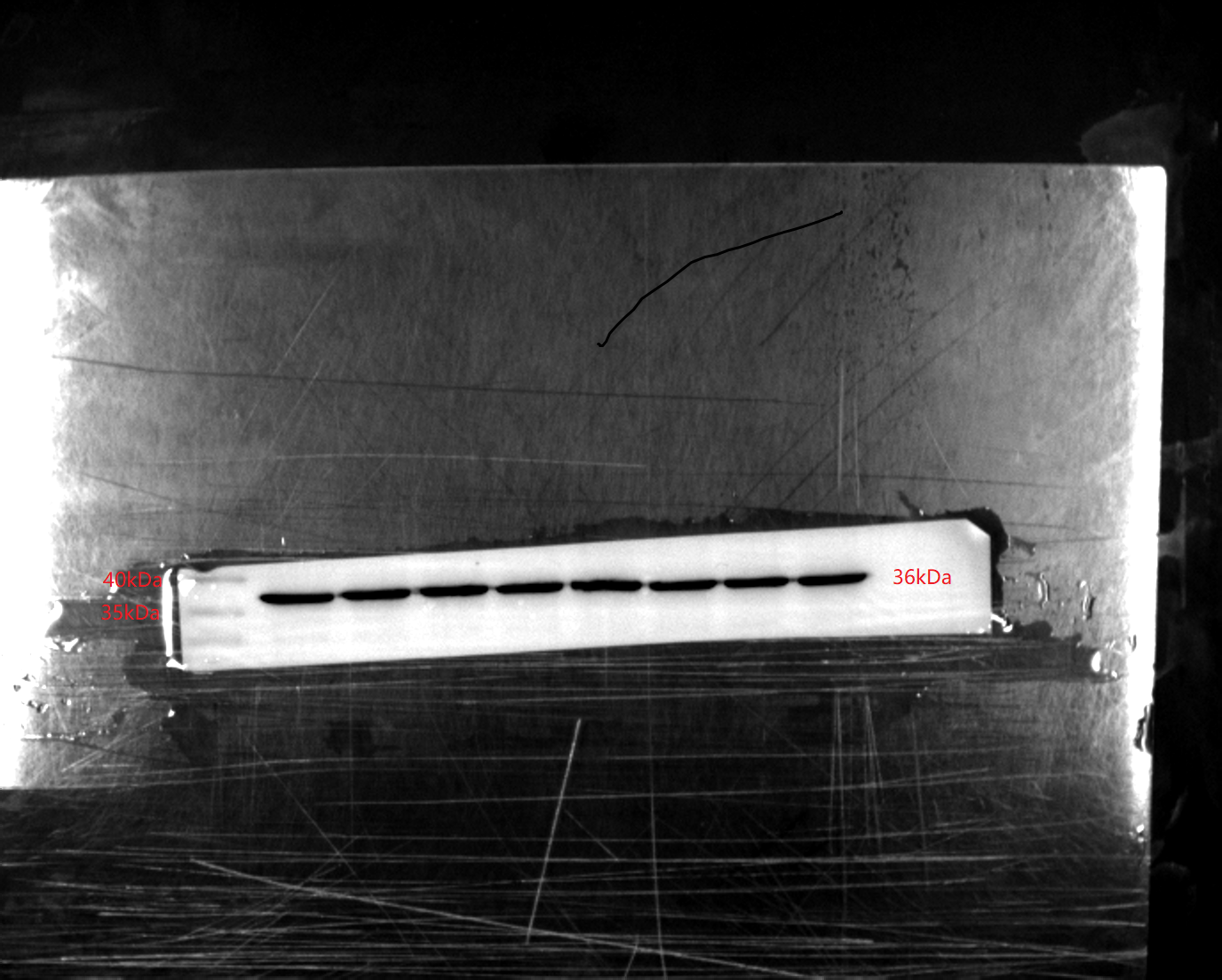

Supplement: Supplemental Information 1 [file peerj-11-15706-s001.zip › Full-length uncropped gelsblots/GAPDH-1-1.Tif]

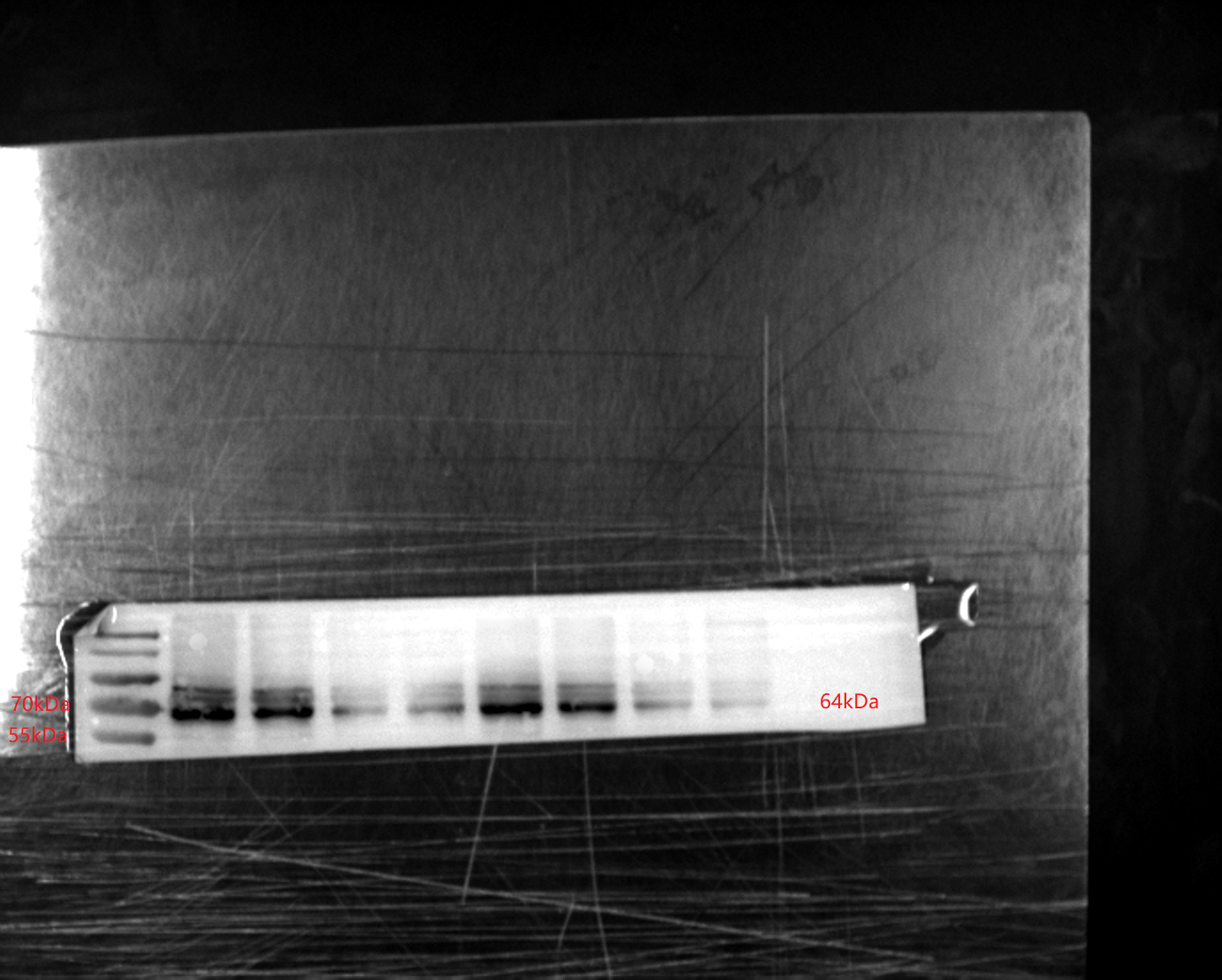

Supplement: Supplemental Information 1 [file peerj-11-15706-s001.zip › Full-length uncropped gelsblots/IGF2BP3-1-1.Tif]

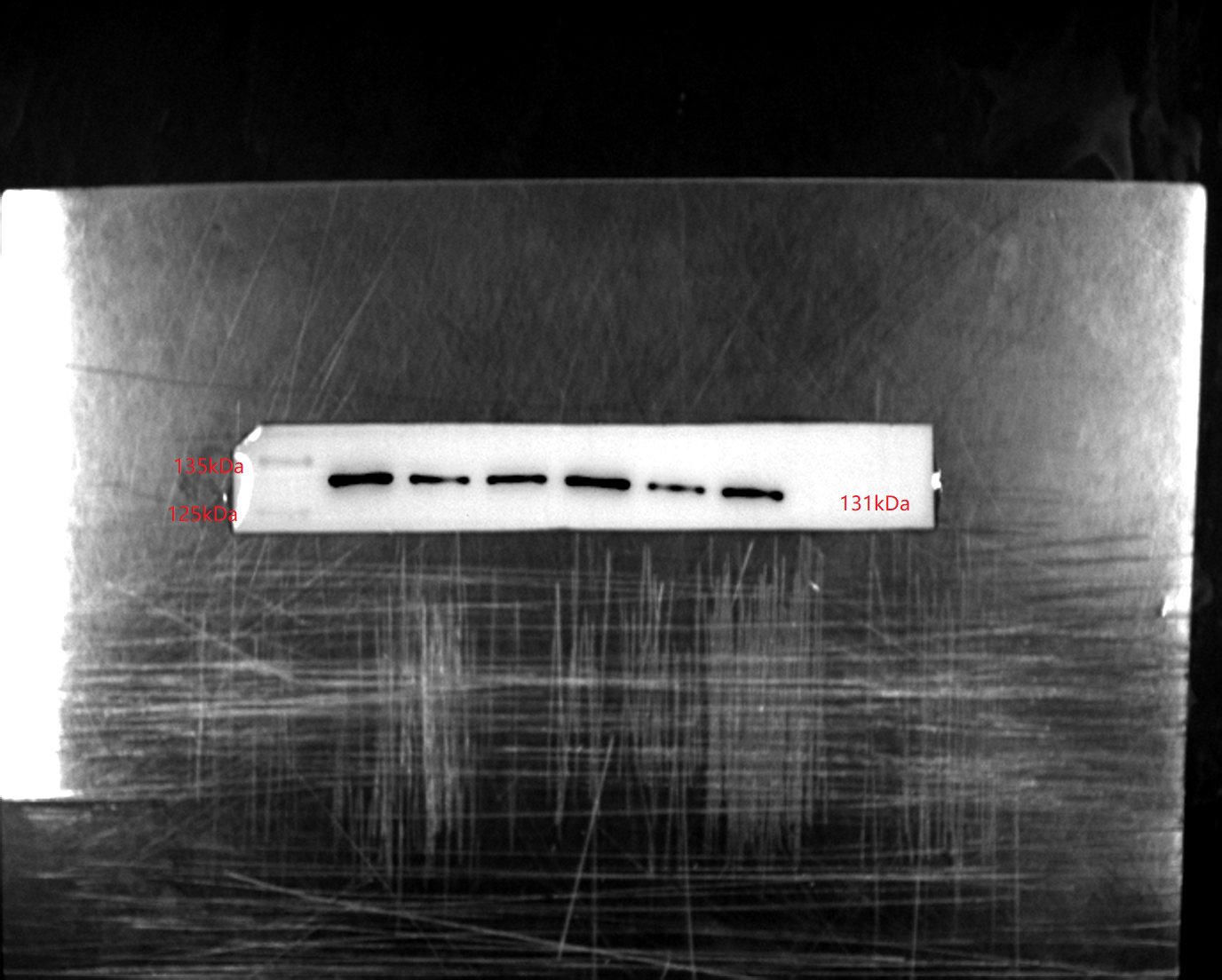

Supplement: Supplemental Information 1 [file peerj-11-15706-s001.zip › Full-length uncropped gelsblots/p-JAK2.Tif]

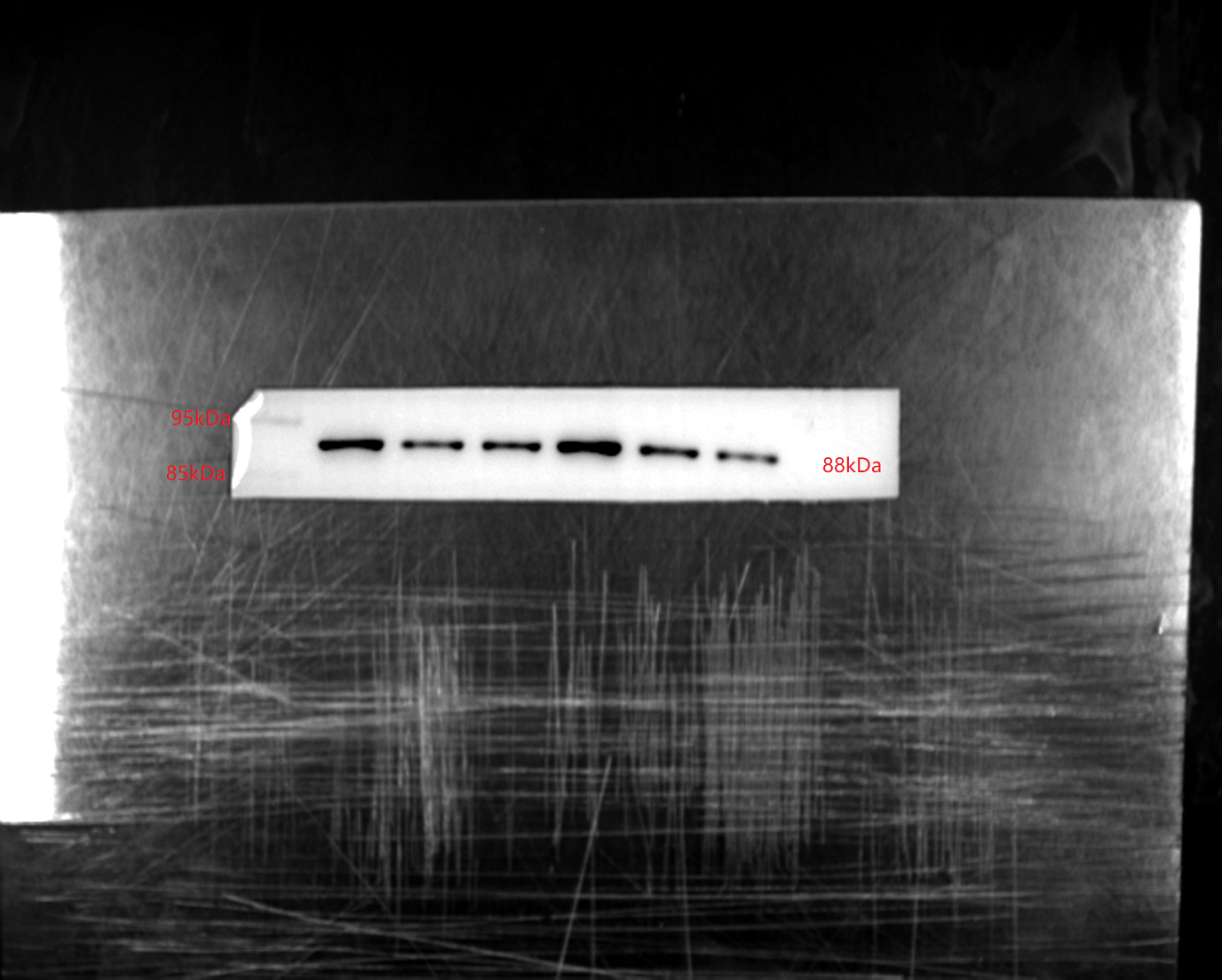

Supplement: Supplemental Information 1 [file peerj-11-15706-s001.zip › Full-length uncropped gelsblots/p-STAT5.Tif]
